# Using laxative to recover intestinal function could improve the survival outcome for patients with cardiac arrest: a retrospective cohort study from MIMIC-IV database

**DOI:** 10.1186/s40560-026-00861-y

**Published:** 2026-02-09

**Authors:** Jingwei Duan, Yuanchao Shi, Yufan Du, Dilu Li, Baomin Duan, Guoxing Wang

**Affiliations:** 1 Department of Emergency, Beijing Friendship Hospital, Capital Medical University, 95th Yong’an Road, Xicheng District, Beijing, China; 2 Department of Medical Oncology, Beijing Friendship Hospital, Capital Medical University, Beijing, China; 3 Department of Emergency, Kaifeng Central Hospital, Kaifeng, China

**Keywords:** Cardiac arrest, Laxative, Intestinal function, Survival outcome, MIMIC-IV database

## Abstract

**Background:**

Despite advances in post-cardiac arrest (CA) care, mortality rates remain high. Intestinal dysfunction following CA is associated with adverse clinical outcomes. While laxatives might be a potential therapeutic strategy for restoring intestinal function, there is currently a lack of evidence.

**Objective:**

This study aims to explore the safety and efficacy of laxatives as therapeutic agents for intestinal function recovery in CA, while further assessing the most suitable types of laxatives for this clinical context.

**Methods:**

Utilizing the MIMIC-IV database, we conducted a retrospective cohort study categorizing patients into laxative and non-laxative groups. Propensity score matching (PSM) was applied to balance baseline characteristics. We classified confounders into prehospital and hospitalization categories, establishing three hierarchical adjustment models. Through multivariable Cox and logistic regression analyses, we assessed 30-day and extended mortality risks while evaluating laxative-associated safety outcomes. Time-dependent Cox regression analysis was utilized to assess the time-varying effect of laxative administration. Furthermore, we also comparatively analyzed the risk–benefit profiles of commonly used laxatives.

**Results:**

2604 patients were eligible for this study. After PSM, a total of 898 patients were average divided into two groups. Patients in the laxative group demonstrated a statistically significant reduction in 30-day mortality risk compared to the non-laxative group (HR = 0.686, 95%CI [0.550–0.855], *P* = 0.001). After applying time-dependent Cox regression analysis, the results remain consistent (HR = − 0.771 + 0.632 × ln[day + 1], 95%CI [0.528–0.835], *P* = 0.001). Meanwhile, laxative did not increase the risk of sepsis and Clostridium difficile infection but improved the bowel sounds recovery and increased the ICU-free day. Further subgroup analysis revealed that the use of docusate sodium (HR = 0.753, 95%CI [0.566–1.000], *P* = 0.050) may be associated with a decreased mortality risk.

**Conclusion:**

Early using laxative to improve intestinal function is associated with improved survival outcomes. However, clinical application may be guided by the bowel sounds recovery and demonstrated tolerance to enteral feeding. This still requires further studies to confirm.

**Supplementary Information:**

The online version contains supplementary material available at 10.1186/s40560-026-00861-y.

## Introduction

Since the last guidelines were published, cardiac arrest (CA) remains one of the leading causes of mortality worldwide [1]. Despite advances in post-CA care, the mortality rate remains high. Based on the latest statistics, survival to hospital discharge was 7.5–10.5% for patients with out-of-hospital cardiac arrest (OHCA), and 23.6–35% for in-hospital cardiac arrest (IHCA) [2, 3]. Meanwhile, the morbidity of CA is rising year by year, placing a growing number of individuals at risk in the future [3]. Therefore, improving survival rates for CA patients remains a critical issue that urgently requires exploration.

A recent study demonstrated that intestinal injury was associated with multiple organ dysfunction [4]. This not only highlights the need to focus on the recovery of intestinal function after CA but also underscores numerous overlooked aspects in post-cardiac arrest care. Following CA, the combination of severe intestinal ischemia and hypoxia with subsequent persistent hypoperfusion due to high-dose vasoactive agents often leads to intestinal dysfunction, frequently manifesting as feeding intolerance caused by impaired motility [5, 6]. Concurrently, targeted temperature management strategies following CA may further exacerbate pre-existing intestinal dysfunction [7]. While temporarily diverting attention and resources away from the gut to protect vital organs like the heart, brain, and kidneys may seem justifiable in the short term, prolonged practice can lead to intestinal microbiota dysbiosis, subsequently triggering Clostridium difficile (C-diff) infection, bacterial translocation, and coagulation disorders [8–10]. Meanwhile, a recent study showed that early enteral nutrition could improve clinical outcomes for patients with CA [11]. Therefore, we notice that maintaining intestinal function seems to improve clinical outcomes for CA.

Previous views showed that laxatives are commonly employed to address constipation and preserve intestinal function [12]. These agents work by altering the physical or chemical properties of intestinal contents or by directly stimulating peristalsis [13]. Commonly used laxatives currently include senna (stimulants), lactulose (osmotic agents), polyethylene glycol (osmotic agents), and docusate sodium (stimulants), each exerting its laxative effects through distinct mechanisms [14]. However, historical clinical experience shows that concerns persist regarding laxative use, particularly their potential to increase the risk of C-diff infection and exacerbate fluid and electrolyte imbalances, among other associated risks [15, 16]. However, due to the absence of relevant studies, we are unable to evaluate the potential benefits and risks of laxative use following CA.

This study leverages the Medical Information Mart for Intensive Care (MIMIC)-IV database to assess the clinical outcomes and safety profiles of laxative use versus non-use following CA, while also evaluating the efficacy and safety of specific laxative classes.

## Methods

### Data sources and informed consent

We conducted this retrospective cohort study from the latest version of MIMIC-IV (v. 3.1) database. This database is a publicly available, de-identified dataset and encompasses comprehensive records of patients admitted to the emergency department or intensive care unit at the Beth Israel Deaconess Medical Center in Boston, MA from 2008 to 2022 [17, 18]. From this database, we can obtain patient characteristics, vital signs, laboratory results, comorbidities, treatments, and clinical outcomes, allowing for analyses of critically ill populations. Before accessing the database, mandatory certification in Protecting Human Research Participants must be obtained. The first author, Jingwei Duan, of this study has obtained this certification (No. 36003834) and secured approval for database access. All data in this database were de-identified, leading the Ethics Committee of Beth Israel Deaconess Medical Center to grant a waiver of informed consent. Furthermore, this study adheres to the STROBE statement and the Declaration of Helsinki.

### Inclusion and exclusion criteria

This study has the following inclusion criteria: (a) patients with CA; (b) age ≥ 18 years; (c) any initial rhythm; (d) OHCA and IHCA.

**Fig. 1:**
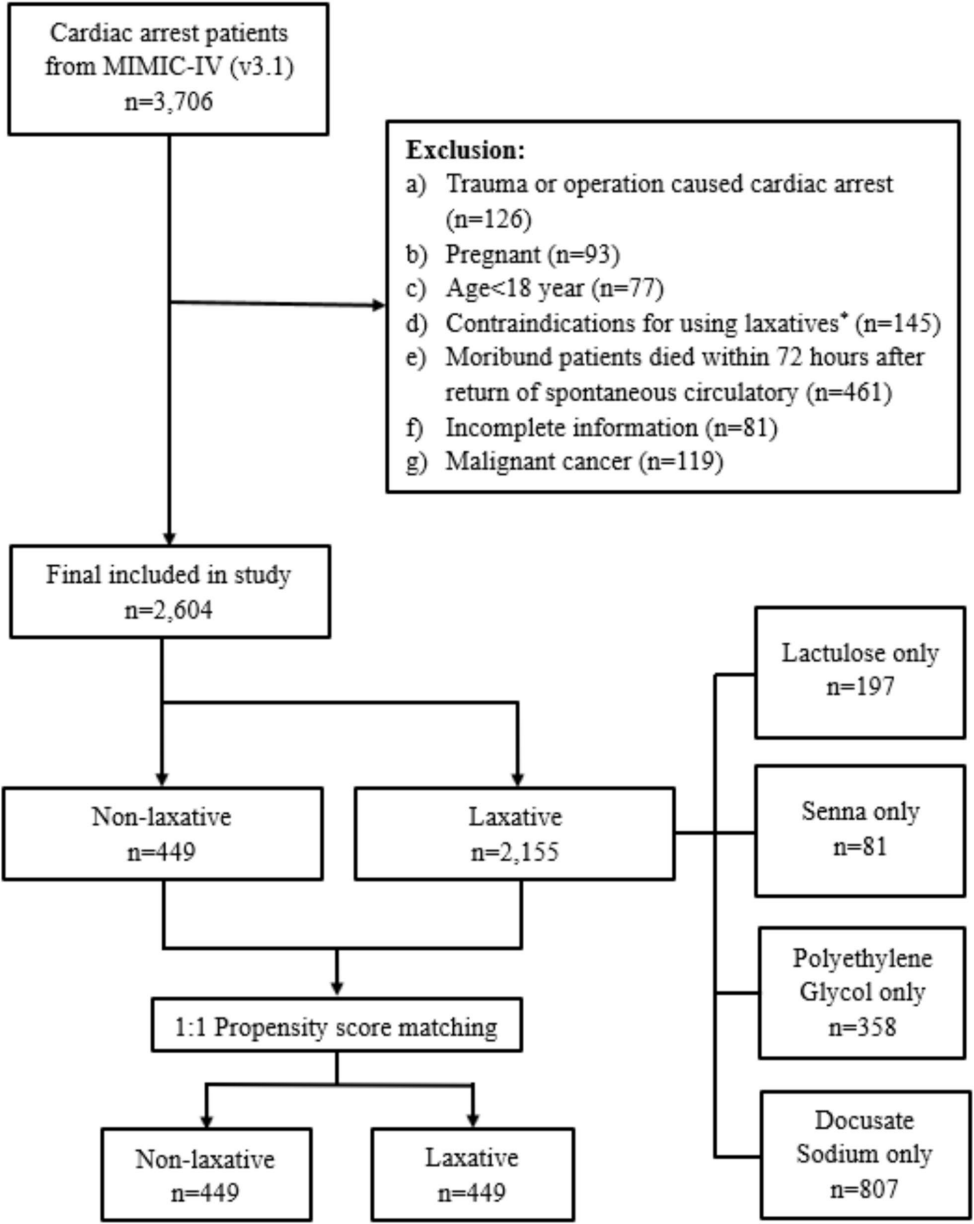
Flowchart. *The contraindications for using laxatives include gastrointestinal hemorrhage, intestinal obstruction, and allergy to laxatives

Exclusion criteria: (a) pregnant; (b) CA caused by trauma or operation; (c) patients with any contraindication for using laxative (including: gastrointestinal hemorrhage, intestinal obstruction, and allergy to laxatives); (d) moribund patients who died within 48 h of return of spontaneous circulation (ROSC); (e) with malignant or severe disease and life expectancy less than 3 months (e.g., malignant tumor or severe stroke); (f) more than 20% of missing data were included (Fig. 1).

### Endpoints definition

The primary endpoint was 30-day cumulative all-cause mortality after ROSC. The secondary endpoints were as follows: (a) 90-day cumulative all-cause mortality after ROSC; (b) 180-day cumulative all-cause mortality after ROSC; (c) using cerebral performance categories (CPC) 3–5 to represent bad neurologic outcome at discharge; (d) ICU-free day. And the safety endpoints were as follows: (a) C-diff infection; (b) unrecovered bowel sounds; (c) sepsis. All safety endpoints were defined as events occurring during hospitalization.

### Data extraction

Firstly, we initially identified CA patients through International Classification of Diseases (ICD) codes. To mitigate potential case omission from relying solely on ICD coding, we additionally reviewed electrocardiographic readings and monitoring data consistent with cardiac arrest manifestations, thereby ensuring more comprehensive patient enrollment. Based on the design of previous classic studies and other readily accessible potential intervention factors [11, 19], we conducted data extraction for enrolled patients by using PostgreSQL (v. 17.0). The extracted data were divided into three parts of prehospital characteristics (demographics, comorbidities, and CA characteristics), hospitalization variables (vital signs, treatments, and risk scores), and endpoints (primary, secondary, and safety endpoints). To minimize bias associated with relying on a single risk assessment tool, we incorporated multiple established risk scoring systems including Sequential Organ Failure Assessment (SOFA), Simplified Acute Physiology Score II (SAPS II), Acute Physiology Score III (APS III), and Oxford Acute Severity of Illness Score (OASIS). Additionally, the Charlson comorbidity index was employed to account for potential confounding effects of pre-existing conditions. All laboratory variables and disease severity scores were derived from the first recorded data after ROSC. The in-hospital characteristics specified in Model 2 were all recorded within 24 h after ROSC. Specifically: vital signs were recorded immediately following ROSC; blood gas analysis results reflect the first measurement after ROSC; the use of coronary angiography/percutaneous coronary intervention, continuous renal replacement therapy, and invasive mechanical ventilation was documented based on whether they were administered within 24 h post-ROSC; medication use refers to drugs given within 24 h after ROSC; while the Vasoactive-Inotropic Score (VIS), SOFA score, SAPS-II, APS-III, and OASIS were recorded as the worst values observed within the first 24 h after ROSC.

### Statistical analysis

All continuous variables are presented as the median and interquartile range (IQR). Since all continuous variables in this study exhibited non-normal distributions, categorical variables were presented for total number and percentage. Mann–Whitney U test was used to compare continuous variables, and *X*^2^ test or Fisher’s exact test was used to compare categorical variables.

Kaplan–Meier (K-M) curves were used to show cumulative survival probabilities, and log-rank tests were conducted to compare cumulative survival distributions between groups. Multivariable Cox regression models were used to adjust for the impact of confounding factors on cumulative survival endpoints. Meanwhile, multivariable logistic regression was utilized to account for confounding factors affecting non-cumulative endpoints. Differences in ICU-free days were analyzed using linear regression, with adjustments applied according to Model 1, 2, and 3. The results are presented as adjusted mean differences along with their corresponding 95% confidence intervals.

Based on prior experience, we have constructed three adjusted models to control for confounding effects: Model 1 adjusted for prehospital characteristics (including gender, age, BMI, smoke, race, comorbidities, Charlson comorbidity index, OHCA, lay securer CPR, and initial rhythm shockable), Model 2 adjusted for hospitalization characteristics (including ECPR, vital signs, hypothermia, invasive ventilator, CAG/PCI, CRRT, MCS, early enteral nutrition, medications, vasoactive inotropic score, and risk scoring), and Model 3 adjusted for prehospital and hospitalization characteristics. All covariates included in the Cox regression models were tested for adherence to the proportional hazards assumption using Schoenfeld residual analysis. The assessment revealed that the variables "laxative use or not", "MAP", and "heart rate" violated the proportional hazards assumption, while all other covariates satisfied it. Following standard methodology, MAP and heart rate were stratified for analysis: MAP was dichotomized at ≥ 60 mmHg and < 60 mmHg, and heart rate was stratified at > 100 bpm and ≤ 100 bpm (Supplementary Table 7). Since the use of laxatives is a key covariate in this study and exhibits a typical time-dependent effect, we additionally performed a time-dependent Cox model analysis. For the time-varying effect of laxatives, considering that its impact may be pronounced early on and stabilize later, we modeled it using a log-linear function of time (ln(day + 1)*covariate).

Patients were categorized into laxative and non-laxative groups based on whether they received laxative treatment. To minimize confounding effects from intergroup characteristic differences, we conducted 1:1 propensity score matching (PSM) to balance baseline characteristics. PSM was performed using caliper matching, with the caliper width set at 0.2 times the standard deviation of the propensity scores for both groups. The calculated caliper value for this study was determined to be 0.015. Initially, Kaplan–Meier curves were utilized to illustrate survival distributions. Subsequently, Models 1, 2, and 3 were applied to adjust for potential confounding factors.

To further investigate which laxative may be more suitable for managing intestinal dysfunction in CA, we categorized cases based on four clinically common laxatives (senna, lactulose, polyethylene glycol, and docusate sodium) and conducted a comparative analysis focusing exclusively on patients administered any single agent from these categories. The Kruskal–Wallis test was used to compare continuous variables in the comparative analysis. Given the limited number of patients receiving either lactulose or senna alone, performing four-group PSM would result in a significant loss of statistical power. Therefore, we conducted multivariable Cox or logistic regression analyses solely on the crude cohort, and the senna group was the reference.

Additional sensitivity analyses were performed using Model 3 multivariable Cox regression to evaluate 30-day cumulative survival, aiming to assess potential benefits in specific subpopulations.

A two-tailed *P*-value of less than 0.05 was considered statistically significant. All statistical analyses were performed using SPSS version 25.0 (SPSS Inc., Chicago, IL, USA).

## Results

### Baseline characteristics

From initially identified 3,706 patients, a total of 2,604 patients were eligible for this study. Of these eligible patients, 449 patients (17.2%) were treated without laxative and 2155 patients (82.8%) with laxative. Significant statistical differences were observed between the two groups in terms of prehospital and hospitalization characteristics and disease severity. The cohort had a lower proportion of females compared to males (35.4% vs. 64.6%), with no significant difference in gender distribution between the two groups. The SAPS-II scores were significantly lower in the laxative group compared to the non-laxative group (41 [31–52] vs. 43 [33–54], *P* = 0.039), while the OASIS scores were notably higher in the laxative group (35 [30–42] vs. 35 [28–41], *P* = 0.003). This suggests that a single risk assessment system may not adequately reflect patients’ disease severity. Patients in the laxative group were more likely to receive early enteral nutrition (34.9% vs. 24.3%, *P* < 0.001), suggesting potentially more favorable intestinal conditions in this cohort (Table 1).

**Table 1:** Original baseline characteristics

	Total (*n* = 2604)	Laxative (*n* = 2155)	Non-laxative (*n* = 449)	*P*-value
Female, *n* (%)	922 (35.4)	776 (36.0)	146 (32.5)	0.175
Age, year, median (IQR)	67 (56–77)	67 (54–77)	65 (54–77)	0.086
BMI, kg/m^2^,	30 (26–32)	30 (26–32)	29 (26–31)	0.023
Smoke, *n* (%)	635 (24.4)	528 (24.5)	107 (23.8)	0.407
Race				0.404
White, *n* (%)	1572 (60.4)	1290 (59.9)	282 (62.8)	
Black, *n* (%)	295 (11.3)	251 (11.6)	44 (9.8)	
Other, *n* (%)	737 (28.3)	614 (28.5)	127 (27.4)	
Comorbidities				
Heart failure, *n* (%)	1235 (47.4)	1016 (47.1)	219 (48.8)	0.282
Myocardial infarction, *n* (%)	928 (35.6)	773 (35.9)	155 (34.4)	0.313
Stroke^*^, *n* (%)	377 (14.5)	326 (15.1)	51 (11.4)	0.021
Diabetes mellitus, *n* (%)	925 (35.5)	770 (35.7)	155 (34.5)	0.334
Renal dysfunction, *n* (%)	752 (28.9)	624 (29.0)	128 (28.5)	0.449
Charlson comorbidity index, median (IQR)	5 (3–7)	5 (3–7)	5 (3–8)	0.524
OHCA, *n* (%)	2020 (77.6)	1666 (77.3)	354 (78.8)	0.260
Lay rescuer CPR, *n* (%)	367 (14.1)	307 (14.2)	60 (13.4)	0.343
Initial rhythm shockable, *n* (%)	1030 (39.6)	859 (39.6)	172 (38.3)	0.289
ECPR, *n* (%)	72 (2.8)	70 (3.2)	2 (0.4)	< 0.001
Heart rate, bpm, median (IQR)	83 (73–96)	83 (73–96)	85 (72–97)	0.580
MAP, mmHg, median (IQR)	79 (73–86)	78 (73–86)	80 (74–87)	0.008
Lactate, mmol/L, median (IQR)	3.7 (2.3–5.0)	3.7 (2.2–4.9)	3.7 (2.4–5.2)	0.544
pH, median (IQR)	7.28 (7.24–7.33)	7.28 (7.24–7.33)	7.28 (7.22–7.32)	0.353
Hypothermia, *n* (%)	316 (12.1)	249 (11.6)	67 (14.9)	0.030
Invasive ventilator, *n* (%)	1537 (59.0)	1248 (57.9)	289 (64.4)	0.006
Coronary arteriography or percutaneous coronary intervention, *n* (%)	150 (5.8)	127 (5.9)	23 (5.1)	0.305
CRRT, *n* (%)	283 (10.9)	233 (10.8)	50 (11.1)	0.448
MCS^#^, *n* (%)	228 (8.8)	201 (9.3)	27 (6.0)	0.013
Early enteral nutrition^$^, *n* (%)	861 (33.1)	752 (34.9)	109 (24.3)	< 0.001
Medications				
Epinephrine, *n* (%)	423 (16.2)	369 (17.1)	54 (12.0)	0.004
Norepinephrine, *n* (%)	1166 (44.8)	964 (44.7)	202 (45.0)	0.481
Dopamine, *n* (%)	274 (10.5)	229 (10.6)	45 (10.0)	0.389
Dobutamine, *n* (%)	452 (17.4)	377 (17.5)	75 (16.7)	0.373
Milrinone, *n* (%)	137 (5.3)	126 (5.8)	11 (2.4)	0.001
Muscle relaxants, *n* (%)	303 (11.6)	242 (11.2)	61 (13.6)	0.092
Vasoactive inotropic score, median (IQR)	4 (0–13)	4 (0–13)	4 (0–13)	0.916
SOFA, median (IQR)	7 (3–10)	7 (3–10)	7 (4–10)	0.769
SAPS-II, median (IQR)	42 (31–52)	41 (31–52)	43 (33–54)	0.039
APS-III, median (IQR)	51 (36–69)	50 (36–69)	54 (38–69)	0.403
OASIS, median (IQR)	35 (28–41)	35 (30–42)	35 (28–41)	0.003

APS-III: Acute Physiology Score III; BMI: body mass index; CPR: cardiopulmonary resuscitation; CRRT: continuous renal replacement therapy; ECPR: extracorporeal cardiopulmonary resuscitation; IQR: interquartile range; MAP: mean arterial pressure; MCS: mechanical circulation support; SAPS-II: Simplified Acute Physiology Score II; SOFA: Sequential Organ Failure Assessment; OASIS: Oxford Acute Severity of Illness Score; OHCA: out-of-hospital cardiac arrest

^*^Stroke includes ischemic and hemorrhagic stroke

^#^MCS includes intra-aortic balloon pump, TandemHeart, Impella, and extracorporeal membrane oxygenation

^$^Early enteral nutrition means patients receiving enteral nutrition within 48 h after return of spontaneous circulation

After performing 1:1 PSM, a total of 898 eligible patients were included, with 449 patients in each group. And no statistically significant difference was observed in all baseline characteristics between the two groups (Supplementary Table 1 and 6).

### Primary endpoint

**Fig. 2:**
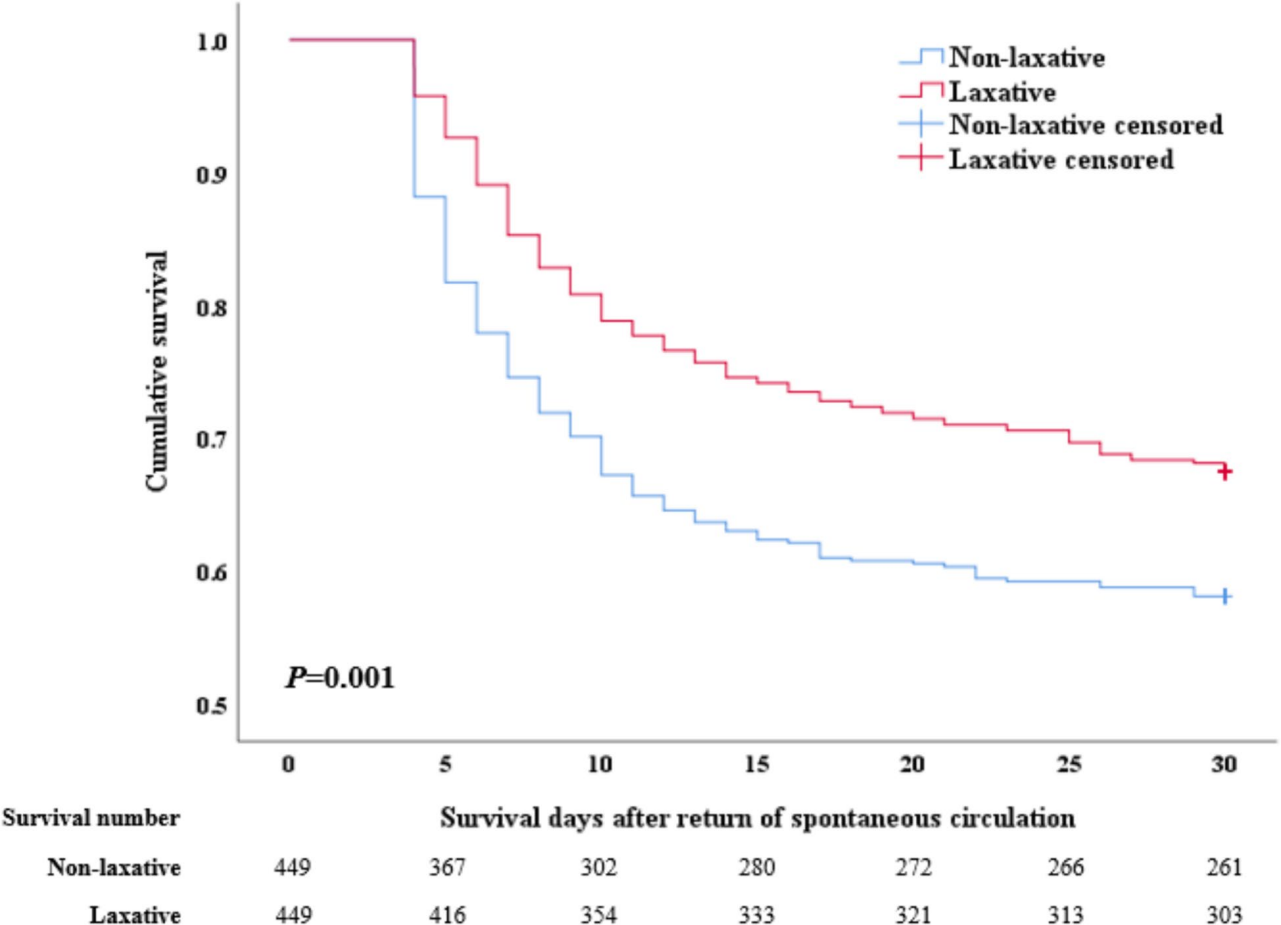
Kaplan–Meier curve for 30-day survival

The K-M curves demonstrated a significant survival advantage in the laxative group (Fig. 2), with superior cumulative 30-day survival outcomes compared to the non-laxative group (HR = 0.697, 95%CI [0.562–0.866], *P* = 0.001). After adjusting for prehospital and hospitalization characteristics, similar findings were observed (HR = 0.686, 95%CI [0.550–0.855], *P* = 0.001). Given the time-dependent nature of laxative use following ROSC, a time-dependent Cox regression analysis was employed to calculate the adjusted HR (HR = -0.771 + 0.632 × ln[day + 1], 95%CI [0.528–0.835], *P* = 0.001). This highlights the critical role of promoting intestinal function recovery in improving survival outcomes for CA patients (Table 2).

**Table 2:** Crude, propensity score matching, and multivariable analyses of using laxative and mortality

	Hazard ratio	95% CI	*P*-value
Crude univariate analysis			
30-day cumulative mortality	0.663	0.564–0.779	< 0.001
90-day cumulative mortality	0.713	0.609–0.835	< 0.001
180-day cumulative mortality	0.715	0.611–0.838	< 0.001
After PSM univariate analysis			
30-day cumulative mortality	0.697	0.562–0.866	0.001
90-day cumulative mortality	0.743	0.603–0.917	0.005
180-day cumulative mortality	0.743	0.603–0.917	0.005
After PSM multivariate analysis			
Model 1^*^			
30-day cumulative mortality	0.723	0.582–0.899	0.004
90-day cumulative mortality	0.766	0.620–0.945	0.013
180-day cumulative mortality	0.766	0.620–0.945	0.013
Model 2^#^			
30-day cumulative mortality	0.665	0.534–0.828	< 0.001
90-day cumulative mortality	0.701	0.567–0.867	0.001
180-day cumulative mortality	0.701	0.567–0.867	0.001
Model 3^$^			
30-day cumulative mortality	0.632	0.550–0.855	0.001
90-day cumulative mortality	0.723	0.584–0.896	0.003
180-day cumulative mortality	0.723	0.584–0.896	0.003
Time-dependent Cox regression for the 30-day cumulative mortality	− 0.771 + 0.632 × ln(day + 1)^^^	0.528–0.835	0.001

^*^Model 1 adjusts prehospital variables which include gender, age, BMI, smoke, race, comorbidities, Charlson comorbidity index, OHCA, lay securer CPR, and initial rhythm shockable

^#^Model 2 adjusts hospitalization variables which include ECPR, vital signs, hypothermia, invasive ventilator, CAG/PCI, CRRT, MCS, early enteral nutrition, medications, vasoactive inotropic score, and risk scoring

^$^Model 3 adjusts both prehospital and hospitalization variables

^The use of laxatives exhibits a time-dependent effect; therefore, its HR should be adjusted according to the following formula. For instance, if laxatives are administered on the third day, the adjusted HR would be 0.105

### Secondary endpoints

Regarding extended cumulative survival outcomes, patients receiving laxatives continued to demonstrate superior survival compared to those who did not (HR = 0.743, 95%CI [0.603–0.917], *P* = 0.005 for 90-day and HR = 0.743, 95%CI [0.603–0.917], *P* = 0.005 for 180-day cumulative survival) (Supplementary Fig. 1–2). After adjusting for prehospital and hospitalization characteristics, similar findings were also observed (HR = 0.723, 95%CI [0.584–0.896], *P* = 0.003 for 90-day and HR = 0.723, 95%CI [0.584–0.896], *P* = 0.003 for 180-day cumulative survival) (Table 2).

Regarding neurologic outcome, univariate analysis following PSM initially suggested no significant difference between the two groups at discharge (OR = 0.785, 95%CI [0.604–1.021], *P* = 0.082). However, after full covariate adjustment in Model 3, patients who did not receive laxatives demonstrated significantly worse neurologic outcomes at discharge (OR = 0.663, 95%CI [0.469–0.938], *P* = 0.020). This suggests that strategies aimed at improving intestinal function may enhance neurologic recovery following CA (Table 3).

**Table 3:** Results of secondary and safety endpoints

	Laxative	Non-laxative	Odds ratio	95% CI	*P*-value
**Crude univariate analysis**	**n = 2155**	**n = 449**			
CPC 3–5 at discharge, *n* (%)	956 (44.4)	224 (49.9)	0.801	0.653–0.982	0.033
Sepsis, *n* (%)	382 (17.7)	98 (21.8)	0.836	0.707–0.976	0.026
Unrecovered bowel sounds, *n* (%)	52 (2.4)	23 (5.1)	0.458	0.277–0.756	0.003
C-diff infection, *n* (%)	58 (2.7)	17 (3.8)	0.703	0.405–1.219	0.135
ICU-free day (day)^*^, median (IQR)	5 (1–10)	3 (0–6)	3.180^#^	1.645–4.714	< 0.001
**After PSM univariate analysis**	**n = 449**	**n = 449**			
CPC 3–5 at discharge, n (%)	197 (43.9)	224 (49.9)	0.785	0.604–1.021	0.082
Sepsis, n (%)	71 (15.8)	98 (21.8)	0.673	0.480–0.944	0.013
Unrecovered bowel sounds, n (%)	8 (1.8)	23 (5.1)	0.336	0.149–0.759	0.005
C-diff infection, n (%)	9 (2.0)	17 (3.8)	0.520	0.229–1.179	0.081
ICU-free day (day)^*^, median (IQR)	4 (1–8)	3 (0–6)	1.869^#^	0.453–3.284	0.010
After PSM multivariate analysis					
Model 1					
CPC 3–5 at discharge			0.735	0.543–0.995	0.046
Sepsis			0.675	0.478–0.952	0.025
Unrecovered bowel sounds			0.340	0.147–0.785	0.011
C-diff infection			0.550	0.240–1.259	0.157
ICU-free day (day)^*^			1.902^#^	0488–3.316	0.008
Model 2					
CPC 3–5 at discharge			0.737	0.542–989	0.049
Sepsis			0.631	0.438–0.909	0.013
Unrecovered bowel sounds			0.294	0.126–0.681	0.004
C-diff infection			0.515	0.225–1.181	0.117
ICU-free day (day)^*^			1.815^#^	0.414–3.217	0.011
Model 3					
CPC 3–5 at discharge			0.663	0.469–0.938	0.020
Sepsis			0.623	0.431–0.901	0.012
Unrecovered bowel sounds			0.303	0.128–0.717	0.007
C-diff infection			0.544	0.234–1.262	0.156
ICU-free day (day)^*^			1.929^#^	0.525–3.334	0.007

**Crude univariate analysis**: unadjusted univariate logistic or linear regression analysis was performed on the initially enrolled patients, with no covariate adjustments applied. **After PSM univariate analysis**: propensity score matching was conducted on the initial cohort, followed by unadjusted univariate logistic or linear regression analysis on the matched population without covariate adjustment

^*^ICU-free day was analyzed by using linear regression

^#^Adjusted mean difference

### Safety endpoints

Regarding safety endpoints, this study established four laxative-related adverse outcome measures (sepsis, unrecovered bowel sounds, C-diff infection, and ICU-free day) based on potential adverse effects associated with laxative use. After PSM, univariate analysis demonstrated that laxative use reduced the risk of sepsis (OR = 0.673, 95%CI [0.480–0.944], *P* = 0.013) and unresolved bowel sounds (OR = 0.336, 95%CI [0.149–0.759], *P* = 0.005) without increasing the incidence of C-diff infection (OR = 0.520, 95%CI [0.229–1.179], *P* = 0.081). Meanwhile, the laxative group demonstrated significantly more ICU-free days compared to the non-laxative group (adjusted mean difference = 1.929, 95%CI [0.525–3.334], *P* = 0.007). Following adjustment in Model 3, multivariable logistic regression analysis revealed consistent results (Table 3).

### Sensitivity analysis

**Fig. 3:**
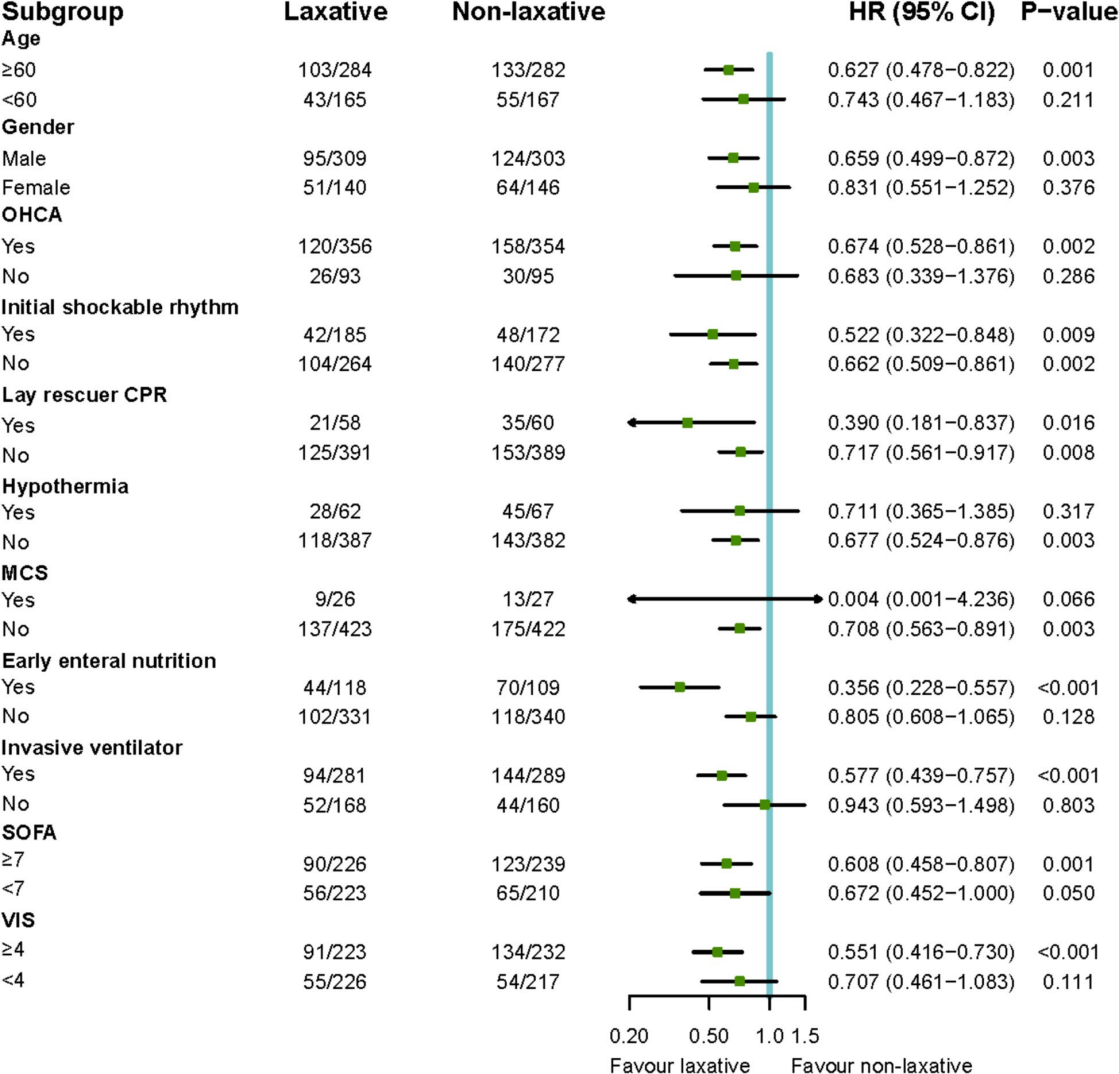
Sensitivity analysis for 30-day mortality. CPR: cardiopulmonary resuscitation; MCS: mechanical circulation support; OHCA: out-of-hospital cardiac arrest; SOFA: Sequential Organ Failure Assessment; VIS: vasoactive inotropic score

Sensitivity analysis was conducted to further investigate whether specific subpopulations would benefit from laxative administration. The results demonstrated statistically significant survival benefits in patients with the following characteristics: age ≥ 60 years, male, OHCA, without hypothermia, without MCS, receipt of early enteral nutrition, treated with invasive mechanical ventilation, SOFA score ≥ 7, and VIS ≥ 4. Furthermore, patients demonstrated consistent benefit from laxative therapy regardless of whether their initial cardiac rhythm was shockable (Fig. 3).

## Results for different laxatives

### Baseline characteristics

A total of 1,443 patients were enrolled in this analysis, with 197 in the senna group, 81 in the lactulose group, 358 in the polyethylene glycol group, and 807 in the docusate sodium group. Significant differences in baseline characteristics were observed among the four groups, notably demonstrating that patients in the lactulose group were younger (60 [52–70]) but more severely ill (the score of SOFA, SAPS II, APS III, and OASIS was significantly higher than the other three groups) (Supplementary Table 2).

### Primary endpoint

Given the limited sample size that would result in substantial loss of statistical power with propensity score matching, we conducted multivariable Cox regression analysis exclusively on the crude cohort. Crude K-M curves revealed that, using the senna group as reference, patients receiving lactulose (HR = 2.348, 95%CI [1.616–3.412], *P* < 0.001) or polyethylene glycol (HR = 1.395, 95%CI [1.032–1.885], *P* < 0.001) showed significantly increased 30-day mortality risk, whereas docusate sodium (HR = 0.956, 95%CI [0.720–1.270], *P* = 0.758) demonstrated no increased mortality risk (Supplementary Fig. 3). Multivariable Cox regression analysis adjusted via Model 3 confirmed the persistence of these findings (HR = 1.575, 95%CI [1.044–2.376], *P* = 0.030 for lactulose; HR = 1.396, 95%CI [1.027–1.898], *P* = 0.033 for polyethylene glycol; HR = 0.836, 95%CI [0.618–1.131], *P* = 0.246 for docusate sodium) (Supplementary Table 3).

### Secondary endpoints

Regarding extended cumulative survival outcomes, K-M curve analysis revealed results consistent with the primary endpoint (Supplementary Fig. 4–5). However, after adjustment via Model 3, multivariable Cox regression demonstrated that neither lactulose (HR = 1.467, 95%CI [0.991–2.172], *P* = 0.056) nor polyethylene glycol (HR = 1.322, 95%CI [0.992–1.762], *P* = 0.057) significantly increased mortality risk, while docusate sodium was associated with significantly improved survival outcomes (HR = 0.753, 95%CI [0.566–1.000], *P* = 0.050) (Supplementary Table 3).

Regarding at-discharge neurologic outcome, after adjustment via Model 3, multivariable logistic regression demonstrated that patients who received lactulose had a significantly increased risk of bad neurologic outcome (OR = 2.361, 95%CI [1.148–4.859], *P* = 0.020), but docusate sodium decreased this risk for patients with CA (OR = 0.534, 95%CI [0.344–0.829], *P* = 0.005) (Supplementary Table 4).

### Safety endpoints

Regarding sepsis, only docusate sodium was associated with a significant reduction in hospital-acquired sepsis (OR = 0.350, 95%CI [0.216–0.566], *P* < 0.001), while the other laxatives showed comparable sepsis risks.

Regarding risk of unrecovered bowel sounds, only lactulose might show increased risk (OR = 14.165, 95%CI [1.496–134.132], *P* = 0.021).

Regarding ICU-free day, compared with senna, docusate sodium significantly increased ICU-free days (adjusted mean difference = 2.450, 95%CI [1.544–4.910], *P* = 0.014), whereas lactulose substantially shortened this duration (adjusted mean difference = -3.290, 95%CI [-2.331- -0.589], *P* = 0.001).

Since no C-diff infection events occurred in the senna group and the other three groups exhibited extremely low incidence rates, further statistical analysis to assess differential risks of C-diff infection was not feasible (Supplementary Table 4).

### Sensitivity analysis

Sensitivity analyses demonstrated that docusate sodium consistently showed potential survival benefits across various subpopulations (OR < 1), whereas lactulose and polyethylene glycol were associated with increased mortality risk in different subpopulations (OR > 1) (Supplementary Table 5).

## Discussion

This study demonstrates that laxatives, as agents for promoting intestinal function recovery, can improve 30-day survival outcomes when administered to CA patients. Previous studies have also indicated that intestinal injury following cardiac arrest is associated with multi-organ dysfunction and increased mortality risk [4].

The intestine plays a crucial role in systemic immune regulation [20, 21]. CA precipitates a state of systemic ischemia and profound hypoxia across all tissues and organs. Following successful CPR and the achievement of ROSC, the body initiates a classic physiological compensatory mechanism whereby intestinal perfusion is markedly reduced to prioritize blood flow to vital organs such as the heart and brain [22, 23]. Concurrently, the administration of high-dose vasoactive agents during post-resuscitation care exacerbates this hemodynamic adaptation, thereby worsening intestinal ischemia [24, 25]. Subsequent to these pathophysiological insults, the intestinal system develops significant functional dysregulation, typically manifested as ischemic necrosis of the intestinal mucosa and markedly diminished motility, ultimately culminating in diarrhea and paralytic ileus [26]. Previous studies have similarly established that early-onset diarrhea following cardiac arrest correlates with unfavorable neurological outcomes, an association attributable to severe intestinal barrier dysfunction and impaired mucosal integrity [27, 28]. Moreover, diminished intestinal motility frequently leads to failure of early enteral feeding, which in turn exacerbates intestinal ischemia and compromises the restoration of normal gut function. Meanwhile, existing research indicates that early enteral nutrition following CA improves clinical outcomes—a conclusion now formally endorsed in the latest guidelines [11, 29]. However, it is crucial to clarify that a prerequisite for successful enteral nutrition is the prior recovery of some degree of intestinal function, and the application of laxatives is one of the key interventions that facilitates this functional recovery. Current evidence indicates a significant correlation between the restoration of intestinal function following CA and favorable clinical outcomes, including improved survival rates and neurological recovery.

Emerging evidence substantiates that intestinal injury following CA precipitates a systemic immune-inflammatory cascade. A prospective cohort study revealed that CA-induced intestinal injury, quantified by plasma intestinal fatty acid-binding protein levels, may mediate systemic immune-inflammatory responses via interleukin-6, thereby triggering subsequent multi-organ injury [30]. Therefore, strategies focusing on intestinal function recovery should be incorporated into post-CA care protocols.

In the context of potent physiological regulatory mechanisms, laxatives currently appear to be the most viable therapeutic option for enhancing intestinal perfusion and restoring gut function. Firstly, laxatives may potentially enhance intestinal blood supply by increasing local prostacyclin or nitric oxide levels, which promotes vasodilation and improves microcirculatory perfusion [31]. Secondary, laxatives can directly stimulate sensory nerve endings in the colonic submucosa, eliciting robust propulsive peristalsis and thereby facilitating the restoration of bowel motility [32]. More importantly, intestinal barrier function is crucial. Our study suggests that laxative use may contribute to improved neurological outcomes in cardiac arrest patients. We hypothesize that the underlying mechanism is related to the gut–brain axis: laxatives likely help maintain intestinal perfusion, thereby supporting the integrity of the gut barrier and preventing the entry of endotoxins, inflammatory mediators, and cytotoxic substances into the bloodstream [33]. The release of these substances can damage the blood–brain barrier and trigger a systemic inflammatory response [34]. This aligns with the observed reduction in sepsis incidence associated with laxative use in the study, as sepsis is fundamentally the result of an excessive inflammatory response leading to multi-organ dysfunction [35]. Therefore, we further speculate that the essential mechanism by which laxatives improve prognosis in CA may be through attenuating systemic inflammation, thereby reducing secondary damage to brain and other organs and tissues. This effect is likely indirect. Since laxatives typically act locally within the intestinal lumen without being systemically absorbed, they may improve intestinal perfusion, thereby helping to maintain gut barrier integrity. This, in turn, could reduce the systemic inflammatory response and mitigate secondary injury to organs and tissues. Furthermore, the effect of laxatives may exhibit time-dependent characteristics. Based on adjustment via the time-effect formula, the HR for laxative administration within 15 days after ROSC is less than 1, indicating that early use of laxatives within this period is more likely to yield clinical benefit. This further underscores that the early recovery of intestinal function and maintenance of gut barrier integrity can effectively improve clinical outcomes in CA patients. Our current findings, while revealing certain clinical correlations, remain observational and necessitate further mechanistic investigation to establish causality.

To further determine the most suitable laxative for post-cardiac arrest care, we evaluated four commonly used agents: senna, lactulose, polyethylene glycol, and docusate sodium. Our analysis revealed that the osmotic laxatives lactulose and polyethylene glycol were associated with a significantly increased 30-day mortality risk in cardiac arrest patients, while senna and docusate sodium demonstrated a neutral or potentially favorable safety profile in multivariable models. The interpretation of these results requires caution due to significant disparities in sample sizes and baseline characteristics between groups—notably the smaller sample size and more severe clinical condition of patients in the lactulose group.

The primary clinical concern regarding laxative use in post-resuscitation patients revolves around their potential hemodynamic impact, particularly the risk that pronounced diarrhea could precipitate or exacerbate intravascular volume depletion, thereby aggravating post-resuscitation shock [36]. We hypothesize that the association between lactulose/polyethylene glycol and poorer clinical outcomes may be attributed to their osmotic mechanisms, which potentially exacerbate intravascular volume depletion through fluid shifts into the intestinal lumen [37]. This could aggravate post-resuscitation shock while simultaneously intensifying intestinal ischemia by potentiating the aforementioned physiological mechanisms [38]. In contrast, senna and docusate sodium exert their laxative effects through direct stimulation of enteric nerves or by lubricating the intestinal wall. This mechanism of action is unlikely to significantly compromise circulatory status, while potentially enhancing intestinal perfusion through increased bowel motility [39].

Consequently, for post-resuscitation patients, laxatives with milder mechanisms of action should be prioritized and administered via careful dose titration starting from minimal doses. The therapeutic objective in this population is not to induce diarrhea but to restore physiological bowel function. Thus, efficacy should not be gauged by the mere induction of diarrhea, but rather by the return of auscultated bowel sounds and demonstrated tolerance to enteral feeding.

Furthermore, this study demonstrated that laxative use did not increase the risk of hospital-acquired sepsis or C-diff infection in CA. Conversely, it was associated with increased ICU-free days and enhanced restoration of bowel sounds. Moreover, both senna and docusate sodium demonstrated a similar trend toward the aforementioned clinical benefits. Our findings, validated through propensity score matching and multiple adjusted regression models, adjust potential confounders, thus supporting their applicability in post-cardiac arrest care practice.

## Limitations

Despite employing multiple analytical approaches, this study still has the following limitations: (a) this study, based on the MIMIC-IV electronic database, is limited by its single-center data source, which may restrict the generalizability of the conclusions; (b) despite attempts to extract supplemental information from free-text entries, the persistent limitations inherent in ICD coding-based data extraction may still result in residual data omissions and biases; (c) the analysis of different laxatives requires cautious interpretation, particularly regarding the lactulose group, due to its limited sample size and greater disease severity at baseline; (d) to minimize the potential impact of critically ill patients on the study outcomes, we excluded those with early mortality (within 72 h after ROSC), thereby limiting the generalizability of our conclusions to patients experiencing severe post-resuscitation conditions; (e) given the absence of out-of-hospital textual data in this database, we were unable to determine the duration of cardiac arrest for OHCA. As this interval is closely associated with patient prognosis, our use of post-ROSC disease severity—assessed via risk stratification scores—as a surrogate measure may introduce some bias into the results; (f) we did not perform time-dependent Cox regression analyses for secondary survival endpoints or sensitivity analyses, which may have led to an overestimation of the effect of laxatives. Nevertheless, based on the findings from the primary survival endpoint analysis, we maintain that early administration of laxatives is more likely to improve clinical outcomes in CA.

## Conclusion

This study demonstrates that using laxatives to improve intestinal function is associated with improved survival outcomes. Similarly, laxative administration appears safe in CA. Regarding the selection of laxatives, greater attention should likely be paid to the actual clinical condition of the patient. The use of lactulose and polyethylene glycol may not provide additional clinical benefit. Guided by enteral feeding tolerance and the recovery of bowel sounds, initiating laxative therapy as early as possible to restore intestinal function may represent a more rational approach. This conclusion remains preliminary and exploratory, necessitating further validation through future multi-center research.

## Supplementary Information

Additional file 1.

## Data Availability

All data of this study extracted from a publicly accessible database. (ohnson, A., Bulgarelli, L., Pollard, T., Gow, B., Moody, B., Horng, S., Celi, L. A., & Mark, R. (2024). MIMIC-IV (version 3.1). PhysioNet. URL: 10.13026/kpb9-mt58). The data that support the findings of this study are available from MIMIC-IV (version 3.1) database but restrictions apply to the availability of these data, which were used under license ( *PhysioNet Credentialed Health Data License 1.5.0*) for the current study, and so are not publicly available. Data are however available from the authors upon reasonable request and with permission of MIT Laboratory for Computational Physiology.

## Abbreviations

APS-III: Acute Physiology Score-III
BMI: Body mass index
CA: Cardiac arrest
C-diff infection: Clostridium difficile infection
CPC: Cerebral performance categories
CPR: Cardiopulmonary resuscitation
CRRT: Continuous renal replacement therapy
ECPR: Extracorporeal cardiopulmonary resuscitation
K-M curve: Kaplan–Meier curve
IHCA: In-hospital cardiac arrest
IQR: Interquartile range
MAP: Mean arterial pressure
MCS: Mechanical circulation support
SAPS-II: Simplified Acute Physiology Score-II
SOFA: Sequential Organ Failure Assessment
OASIS: Oxford Acute Severity of Illness Score
OHCA: Out-of-hospital cardiac arrest
PSM: Propensity score matching
ROSC: Return of spontaneous circulation
