# Supplementary material for: Using laxative to recover intestinal function could improve the survival outcome for patients with cardiac arrest: a retrospective cohort study from MIMIC-IV database
Source: J Intensive Care. 2026 Feb 9;14:28. doi: 10.1186/s40560-026-00861-y (PMC12983495; doi:10.1186/s40560-026-00861-y)
Supplement: Supplementary file 1 — Additional file 1. [file 40560_2026_861_MOESM1_ESM.docx]

**Supplementary table 1.** Baseline characteristics after propensity score matching

|  | **Total (n=898)** | **Laxative (n=449)** | **Non-laxative (n=449)** | ***P*-value** |
| --- | --- | --- | --- | --- |
| Female, n (%) | 286 (31.8) | 140 (31.2) | 146 (32.5) | 0.720 |
| Age, year, median (IQR) | 65 (53-76) | 65 (52-75) | 65 (54-77) | 0.206 |
| BMI, kg/m^2^, | 29 (26-31) | 30 (25-32) | 29 (26-31) | 0.665 |
| Smoke, n (%) | 222 (24.7) | 115 (25.6) | 107 (23.8) | 0.588 |
| Race |  |  |  | 0.739 |
| White, n (%) | 575 (64.0) | 293 (65.3) | 282 (62.8) |  |
| Black, n (%) | 84 (9.4) | 40 (8.9) | 44 (9.8) |  |
| Other, n (%) | 239 (26.6) | 116 (25.8) | 123 (27.4) |  |
| Comorbidities |  |  |  |  |
| Heart failure, n (%) | 439 (48.9) | 220 (49.0) | 219 (48.8) | 1.000 |
| Myocardial infarction, n (%) | 305 (34.0) | 150 (33.4) | 155 (34.5) | 0.778 |
| Stroke^*^, n (%) | 110 (12.2) | 59 (13.1) | 51 (11.4) | 0.476 |
| Diabetes mellitus, n (%) | 323 (36.0) | 168 (37.4) | 155 (34.5) | 0.404 |
| Renal dysfunction, n (%) | 258 (28.7) | 130 (29.0) | 128 (28.5) | 0.941 |
| Charlson Comorbidity Index, median (IQR) | 5 (3-8) | 5 (3-7) | 5 (3-8) | 0.819 |
| OHCA, n (%) | 710 (79.1) | 356 (79.3) | 354 (78.8) | 0.935 |
| Bystander CPR, n (%) | 118 (13.1) | 58 (12.9) | 60 (13.4) | 0.921 |
| Initial rhythm shockable, n (%) | 357 (39.8) | 185 (41.2) | 172 (38.3) | 0.413 |
| ECPR, n (%) | 3 (0.3) | 1 (0.2) | 2 (0.4) | 1.000 |
| Heart rate, bpm, median (IQR) | 85 (73-97) | 84 (74-98) | 85 (72-97) | 0.672 |
| MAP, mmHg, median (IQR) | 80 (74-87) | 80 (74-86) | 80 (74-87) | 0.440 |
| Lactate, mmol/L, median (IQR) | 3.7 (2.4-5.2) | 3.7 (2.3-5.3) | 3.7 (2.4-5.2) | 0.780 |
| pH, median (IQR) | 7.28 (7.23-7.32) | 7.28 (7.23-7.32) | 7.28 (7.22-7.33) | 0.924 |
| Hypothermia, n (%) | 129 (14.4) | 62 (13.8) | 67 (14.9) | 0.704 |
| Invasive ventilator, n (%) | 570 (63.5) | 281 (62.6) | 289 (64.4) | 0.628 |
| Coronary arteriography or percutaneous coronary intervention, n (%) | 51 (5.7) | 28 (6.2) | 23 (5.1) | 0.565 |
| CRRT, n (%) | 101 (11.2) | 51 (11.4) | 50 (11.1) | 1.000 |
| MCS^#^, n (%) | 53 (5.9) | 26 (5.8) | 27 (6.0) | 1.000 |
| Early enteral nutrition^$^, n (%) | 227 (25.3) | 118 (26.3) | 109 (24.3) | 0.539 |
| Medications |  |  |  |  |
| Epinephrine, n (%) | 108 (12.0) | 54 (12.0) | 54 (12.0) | 1.000 |
| Norepinephrine, n (%) | 409 (45.5) | 207 (46.1) | 202 (45.0) | 0.789 |
| Dopamine, n (%) | 88 (9.8) | 43 (9.6) | 45 (10.0) | 0.911 |
| Dobutamine, n (%) | 146 (16.3) | 71 (15.8) | 75 (16.7) | 0.786 |
| Milrinone, n (%) | 25 (2.8) | 14 (3.1) | 11 (2.4) | 0.686 |
| Muscle relaxants, n (%) | 121 (13.5) | 60 (13.4) | 61 (13.6) | 1.000 |
| Vasoactive inotropic score, median (IQR) | 4 (0-12) | 3 (0-12) | 4 (0-13) | 0.412 |
| SOFA score, median (IQR) | 7 (3-10) | 7 (3-9) | 7 (4-10) | 0.578 |
| SAPSII score, median (IQR) | 42 (32-53) | 40 (31-52) | 43 (33-54) | 0.089 |
| APSIII score, median (IQR) | 52 (37-70) | 52 (36-71) | 54 (38-69) | 0.831 |
| OASIS score, median (IQR) | 35 (30-42) | 35 (29-42) | 35 (30-42) | 0.641 |

APS-III: acute physiology score III; BMI: body mass index; CPR: cardiopulmonary resuscitation; CRRT: continuous renal replacement therapy; ECPR: extracorporeal cardiopulmonary resuscitation; IQR: interquartile range; MAP: mean arterial pressure; MCS: mechanical circulation support; SAPS-II: simplified acute physiology score Ⅱ; SOFA: sequential organ failure assessment; OASIS: Oxford acute severity of illness score; OHCA: out-of-hospital cardiac arrest.

^*^stroke includes ischemic and hemorrhagic stroke.

^#^MCS includes intra-aortic ballon pump, TandemHeart, Impella and extracorporeal membrane oxygenation.

^$^early enteral nutrition means patients receiving enteral nutrition within 48 hours after return of spontaneous circulation.

**Supplementary figure 1.** Kaplan-Meier curve 90-day survival


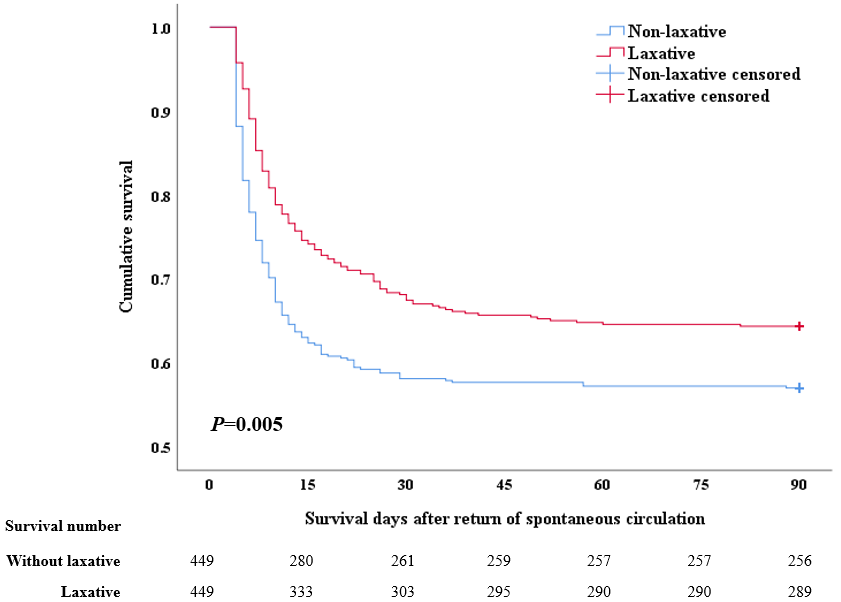


**Supplementary figure 2.** Kaplan-Meier curve for 180-day survival


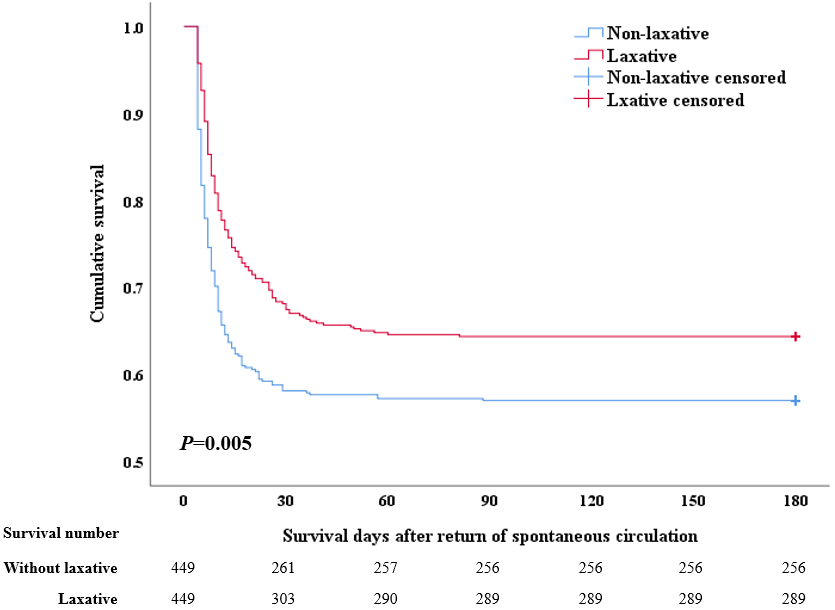


**Supplementary table 2.** Baseline characteristics for different laxatives

|  | **Senna**  **(n=197)** | **Lactulose**  **(n=81)** | **Polyethylene Glycol**  **(n=358)** | **Docusate Sodium**  **(n=807)** | ***P*-value** |
| --- | --- | --- | --- | --- | --- |
| Female, n (%) | 76 (38.6) | 27 (33.3) | 130 (36.3) | 291 (36.1) | 0.856 |
| Age, year, median (IQR) | 67 (54-75) | 60 (52-70) | 68 (58-78) | 68 (56-79) | <0.001 |
| BMI, kg/m^2^, | 30 (26-32) | 30 (28-33) | 29 (26-32) | 30 (26-31) | 0.028 |
| Smoke, n (%) | 49 (24.9) | 24 (29.6) | 88 (24.6) | 191 (23.7) | 0.690 |
| Race |  |  |  |  | <0.001 |
| White, n (%) | 106 (53.8) | 57 (70.4) | 174 (48.6) | 525 (65.1) |  |
| Black, n (%) | 26 (13.2) | 3 (3.7) | 62 (17.3) | 68 (8.4) |  |
| Other, n (%) | 65 (33.0) | 21 (25.9) | 122 (34.1) | 214 (26.5) |  |
| Comorbidities |  |  |  |  |  |
| Heart failure, n (%) | 89 (45.2) | 35 (43.2) | 179 (50) | 366 (45.4) | 0.445 |
| Myocardial infarction, n (%) | 77 (39.1) | 23 (28.4) | 133 (37.2) | 289 (35.8) | 0.385 |
| Stroke^*^, n (%) | 35 (17.8) | 14 (17.3) | 54 (15.1) | 111 (13.8) | 0.477 |
| Diabetes mellitus, n (%) | 68 (34.5) | 28 (34.6) | 116 (32.4) | 295 (36.6) | 0.587 |
| Renal dysfunction, n (%) | 52 (26.4) | 23 (28.4) | 105 (29.3) | 229 (27.6) | 0.891 |
| Charlson Comorbidity Index, median (IQR) | 5 (3-8) | 6 (3-8) | 5 (4-7) | 5 (3-7) | 0.415 |
| OHCA, n (%) | 141 (71.6) | 66 (81.5) | 257 (71.8) | 671 (83.1) | <0.001 |
| Bystander CPR, n (%) | 11 (5.6) | 20 (24.7) | 10 (2.8) | 171 (21.2) | <0.001 |
| Initial rhythm shockable, n (%) | 88 (44.7) | 24 (29.6) | 151 (42.2) | 305 (37.8) | 0.056 |
| ECPR, n (%) | 8 (4.1) | 2 (2.5) | 8 (2.2) | 18 (2.2) | 0.509 |
| Heart rate, bpm, median (IQR) | 84 (73-99) | 93 (83-105) | 82 (73-94) | 81 (72-93) | <0.001 |
| MAP, mmHg, median (IQR) | 80 (74-87) | 75 (70-83) | 79 (74-88) | 79 (73-87) | 0.001 |
| Lactate, mmol/L, median (IQR) | 3.9 (2.6-4.9) | 4.6 (2.8-6.1) | 3.7 (2.5-4.9) | 3.7 (2.1-4.8) | 0.002 |
| pH, median (IQR) | 7.27 (7.24-7.30) | 7.27 (7.21-7.36) | 7.28 (7.25-7.32) | 7.28 (7.23-7.33) | 0.150 |
| Hypothermia, n (%) | 19 (9.6) | 12 (14.8) | 41 (11.5) | 95 (11.8) | 0.663 |
| Invasive ventilator, n (%) | 125 (63.5) | 46 (56.8) | 203 (56.7) | 467 (57.9) | 0.447 |
| Coronary arteriography or percutaneous coronary intervention, n (%) | 3 (1.5) | 2 (2.5) | 4 (1.1) | 84 (10.4) | <0.001 |
| CRRT, n (%) | 25 (12.7) | 12 (14.8) | 34 (9.5) | 78 (9.7) | 0.308 |
| MCS^#^, n (%) | 19 (9.6) | 3 (3.7) | 19 (5.3) | 80 (9.9) | 0.022 |
| Early enteral nutrition^$^, n (%) | 37 (18.8) | 43 (53.1) | 98 (27.4) | 253 (31.4) | <0.001 |
| Medications |  |  |  |  |  |
| Epinephrine, n (%) | 19 (9.6) | 24 (29.6) | 43 (12.0) | 126 (15.6) | <0.001 |
| Norepinephrine, n (%) | 62 (31.5) | 54 (66.7) | 133 (37.2) | 339 (42.0) | <0.001 |
| Dopamine, n (%) | 8 (4.1) | 7 (8.6) | 10 (2.8) | 127 (15.7) | <0.001 |
| Dobutamine, n (%) | 40 (20.3) | 12 (14.8) | 65 (18.2) | 144 (17.8) | 0.736 |
| Milrinone, n (%) | 2 (1.0) | 8 (9.9) | 11 (3.1) | 56 (6.9) | <0.001 |
| Muscle relaxants, n (%) | 9 (4.6) | 6 (7.4) | 20 (5.6) | 120 (14.9) | <0.001 |
| Vasoactive inotropic score, median (IQR) | 0 (0-8) | 10 (0-21) | 0 (0-10) | 4 (0-13) | <0.001 |
| SOFA score, median (IQR) | 7 (4-9) | 10 (8-14) | 6 (4-10) | 6 (2-9) | <0.001 |
| SAPSII score, median (IQR) | 43 (32-52) | 46 (37-59) | 41 (32-53) | 39 (29-50) | <0.001 |
| APSIII score, median (IQR) | 53 (34-72) | 73 (50-91) | 48 (37-63) | 47 (32-65) | <0.001 |
| OASIS score, median (IQR) | 35 (29-42) | 37 (30-45) | 34 (27-41) | 34 (27-40) | 0.007 |

APS-III: acute physiology score III; BMI: body mass index; CPR: cardiopulmonary resuscitation; CRRT: continuous renal replacement therapy; ECPR: extracorporeal cardiopulmonary resuscitation; IQR: interquartile range; MAP: mean arterial pressure; MCS: mechanical circulation support; SAPS-II: simplified acute physiology score Ⅱ; SOFA: sequential organ failure assessment; OASIS: Oxford acute severity of illness score; OHCA: out-of-hospital cardiac arrest.

^*^stroke includes ischemic and hemorrhagic stroke.

^#^MCS includes intra-aortic ballon pump, TandemHeart, Impella and extracorporeal membrane oxygenation.

^$^early enteral nutrition means patients receiving enteral nutrition within 48 hours after return of spontaneous circulation.

**Supplementary figure 3.** Different laxatives Kaplan-Meier curve for 30-day survival


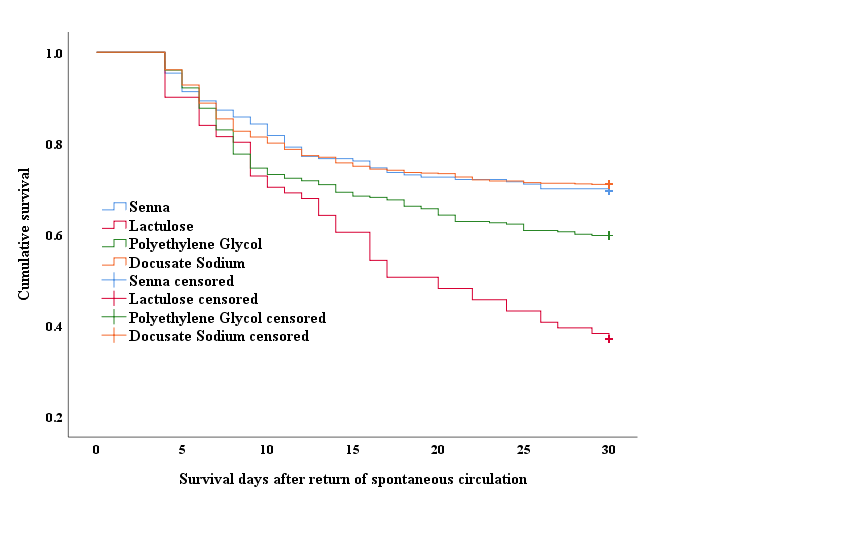


***P*<0.001**

**Supplementary figure 4.** Different laxatives Kaplan-Meier curve for 90-day survival


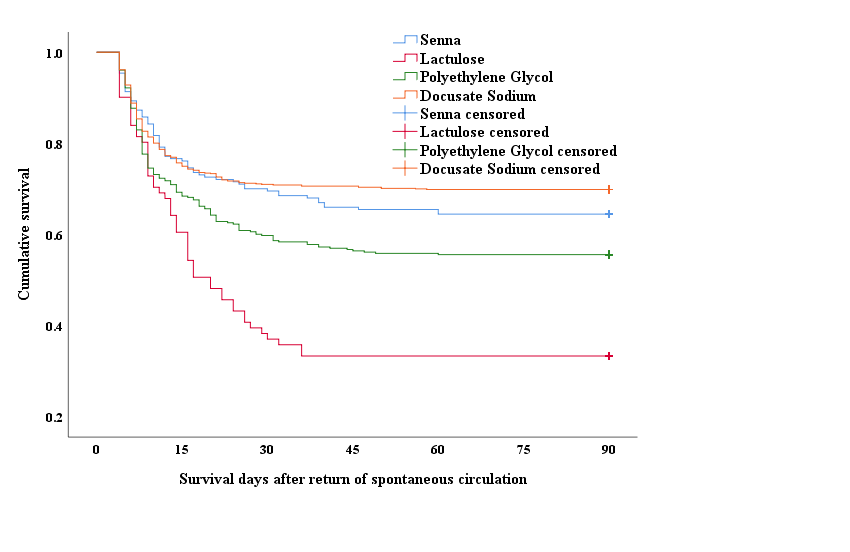


***P*<0.001**

**Supplementary figure 5.** Different laxatives Kaplan-Meier curve for 180-day survival


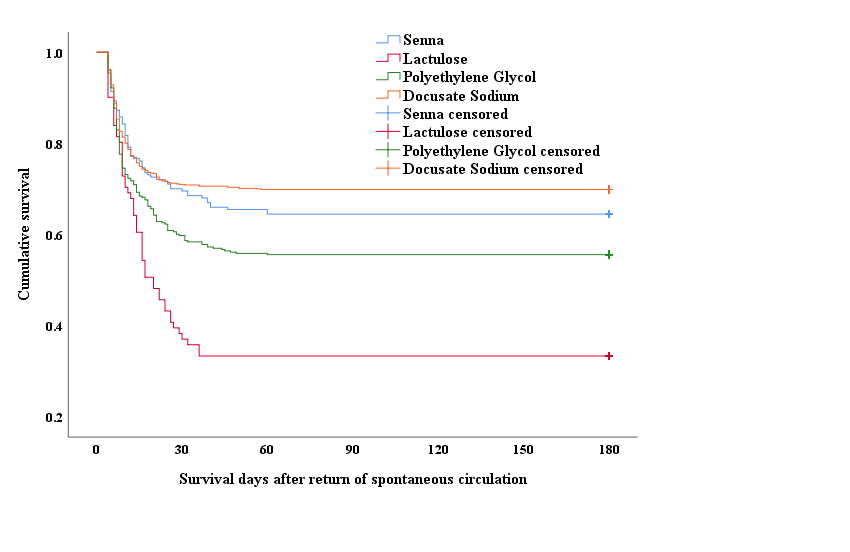


***P*<0.001**

**Supplementary table 3.** Results of mortality risk analysis for different laxatives

|  | **Hazard Ratio** | **95% CI** | ***P*-value** |
| --- | --- | --- | --- |
| **30-day mortality risk** |  |  |  |
| **Univariate analysis** |  |  |  |
| Senna | Ref. | Ref. | Ref. |
| Lactulose | 2.348 | 1.616-3.412 | <0.001 |
| Polyethylene Glycol | 1.395 | 1.032-1.885 | <0.001 |
| Docusate Sodium | 0.956 | 0.720-1.270 | 0.758 |
| **Model 1** |  |  |  |
| Senna | Ref. | Ref. | Ref. |
| Lactulose | 2.270 | 1.550-3.323 | <0.001 |
| Polyethylene Glycol | 1.356 | 1.001-1.839 | 0.049 |
| Docusate Sodium | 0.871 | 0.651-1.166 | 0.353 |
| **Model 2** |  |  |  |
| Senna | Ref. | Ref. | Ref. |
| Lactulose | 1.637 | 1.098-2.441 | 0.016 |
| Polyethylene Glycol | 1.406 | 1.037-1.908 | 0.028 |
| Docusate Sodium | 0.892 | 0.664-1.200 | 0.451 |
| **Model 3** |  |  |  |
| Senna | Ref. | Ref. | Ref. |
| Lactulose | 1.575 | 1.044-2.376 | 0.030 |
| Polyethylene Glycol | 1.396 | 1.027-1.898 | 0.033 |
| Docusate Sodium | 0.836 | 0.618-1.131 | 0.246 |
| **90-day mortality risk** |  |  |  |
| **Univariate analysis** |  |  |  |
| Senna | Ref. | Ref. | Ref. |
| Lactulose | 2.199 | 1.542-3.138 | <0.001 |
| Polyethylene Glycol | 1.330 | 1.004-1.761 | 0.047 |
| Docusate Sodium | 0.848 | 0.650-1.106 | 0.224 |
| **Model 1** |  |  |  |
| Senna | Ref. | Ref. | Ref. |
| Lactulose | 2.165 | 1.506-3.112 | <0.001 |
| Polyethylene Glycol | 1.286 | 0.968-1.708 | 0.083 |
| Docusate Sodium | 0.775 | 0.590-1.019 | 0.068 |
| **Model 2** |  |  |  |
| Senna | Ref. | Ref. | Ref. |
| Lactulose | 1.502 | 1.062-2.199 | 0.037 |
| Polyethylene Glycol | 1.333 | 1.002-1.772 | 0.048 |
| Docusate Sodium | 0.798 | 0.603-1.054 | 0.112 |
| **Model 3** |  |  |  |
| Senna | Ref. | Ref. | Ref. |
| Lactulose | 1.467 | 0.991-2.172 | 0.056 |
| Polyethylene Glycol | 1.322 | 0.992-1.762 | 0.057 |
| Docusate Sodium | 0.753 | 0.566-1.000 | 0.050 |

**Continued supplementary table 3.**

|  | **Hazard Ratio** | **95% CI** | ***P*-value** |
| --- | --- | --- | --- |
| **180-day mortality risk** |  |  |  |
| **Univariate analysis** |  |  |  |
| Senna | Ref. | Ref. | Ref. |
| Lactulose | 2.199 | 1.542-3.138 | <0.001 |
| Polyethylene Glycol | 1.330 | 1.004-1.761 | 0.047 |
| Docusate Sodium | 0.848 | 0.650-1.106 | 0.224 |
| **Model 1** |  |  |  |
| Senna | Ref. | Ref. | Ref. |
| Lactulose | 2.165 | 1.506-3.112 | <0.001 |
| Polyethylene Glycol | 1.286 | 0.968-1.708 | 0.083 |
| Docusate Sodium | 0.775 | 0.590-1.019 | 0.068 |
| **Model 2** |  |  |  |
| Senna | Ref. | Ref. | Ref. |
| Lactulose | 1.502 | 1.026-2.199 | 0.037 |
| Polyethylene Glycol | 1.333 | 1.002-1.772 | 0.048 |
| Docusate Sodium | 0.798 | 0.603-1.054 | 0.112 |
| **Model 3** |  |  |  |
| Senna | Ref. | Ref. | Ref. |
| Lactulose | 1.467 | 0.991-2.172 | 0.056 |
| Polyethylene Glycol | 1.322 | 0.992-1.762 | 0.057 |
| Docusate Sodium | 0.753 | 0.566-1.000 | 0.050 |

Model 1 adjusts prehospital variables which include gender, age, BMI, smoke, race, comorbidities, Charlson Comorbidity Index, OHCA, lay securer CPR and initial rhythm shockable.

Model 2 adjusts hospitalization variables which include ECPR, vital signs, hypothermia, invasive ventilator, CAG/PCI, CRRT, MCS, early enteral nutrition, medications, vasoactive inotropic score and risk scoring.

Model 3 adjusts both prehospital and hospitalization variables.

**Supplementary table 4.** Model 3 adjusted analysis results of secondary and safety endpoints for different laxatives

|  | **Odds ratio** | **95% CI** | ***P*-value** |
| --- | --- | --- | --- |
| **CPC 3-5 at discharge** |  |  |  |
| Senna | Ref. | Ref. | Ref. |
| Lactulose | 2.361 | 1.148-4.859 | 0.020 |
| Polyethylene Glycol | 1.384 | 0.882-2.172 | 0.158 |
| Docusate Sodium | 0.534 | 0.344-0.829 | 0.005 |
| **Sepsis** |  |  |  |
| Senna | Ref. | Ref. | Ref. |
| Lactulose | 0.819 | 0.414-1.617 | 0.565 |
| Polyethylene Glycol | 1.179 | 0.737-1.888 | 0.492 |
| Docusate Sodium | 0.350 | 0.216-0.566 | <0.001 |
| **Unrecovered bowel sounds** |  |  |  |
| Senna | Ref. | Ref. | Ref. |
| Lactulose | 14.165 | 1.496-134.132 | 0.021 |
| Polyethylene Glycol | 0.833 | 0.069-10.111 | 0.886 |
| Docusate Sodium | 3.232 | 0.394-26.600 | 0.275 |
| **ICU-free day (day)^*^** |  |  |  |
| Senna | Ref. | Ref. | Ref. |
| Lactulose | -3.290^#^ | -2.331- -0.589 | 0.001 |
| Polyethylene Glycol | -0.437^#^ | -2.850-1.824 | 0.667 |
| Docusate Sodium | 2.450^#^ | 1.544-4.910 | 0.014 |

^*^ICU-free day waw analyzed by using linear regression.

^#^adjusted mean difference

**Supplementary table 5.** Results of sensitive analysis for different laxatives

| **Subgroup** | **Mortality/total number** | **Hazard ratio** | **95% CI** | ***P*-value** |
| --- | --- | --- | --- | --- |
| **Age, year** |  |  |  |  |
| ≥60 |  |  |  |  |
| Senna | 59/129 | Ref. | Ref. | Ref. |
| Lactulose | 28/41 | 1.457 | 0.869-2.442 | 0.153 |
| Polyethylene Glycol | 108/257 | 1.134 | 0.804-1.600 | 0.473 |
| Docusate Sodium | 179/565 | 0.687 | 0.490-0.964 | 0.030 |
| <60 |  |  |  |  |
| Senna | 10/68 | Ref. | Ref. | Ref. |
| Lactulose | 23/40 | 2.156 | 0.937-4.959 | 0.071 |
| Polyethylene Glycol | 36/101 | 2.610 | 1.209-5.638 | 0.015 |
| Docusate Sodium | 55/242 | 1.209 | 0.540-2.704 | 0.644 |
| **Gender** |  |  |  |  |
| Male |  |  |  |  |
| Senna | 40/121 | Ref. | Ref. | Ref. |

**Continued supplementary table 5.**

| **Subgroup** | **Mortality/total number** | **Hazard ratio** | **95% CI** | ***P*-value** |
| --- | --- | --- | --- | --- |
| Lactulose | 30/54 | 1.281 | 0.756-2.169 | 0.357 |
| Polyethylene Glycol | 92/228 | 1.225 | 0.835-1.796 | 0.300 |
| Docusate Sodium | 134/516 | 0.673 | 0.459-0.989 | 0.044 |
| Female |  |  |  |  |
| Senna | 20/76 | Ref. | Ref. | Ref. |
| Lactulose | 21/27 | 1.772 | 0.852-3.687 | 0.126 |
| Polyethylene Glycol | 52/103 | 1.502 | 0.862-2.615 | 0.151 |
| Docusate Sodium | 100/219 | 1.055 | 0.622-1.789 | 0.151 |
| **OHCA** |  |  |  |  |
| Yes |  |  |  |  |
| Senna | 41/141 | Ref. | Ref. | Ref. |
| Lactulose | 43/66 | 1.510 | 0.941-2.425 | 0.088 |
| Polyethylene Glycol | 101/257 | 1.424 | 0.983-2.065 | 0.062 |
| Docusate Sodium | 199/671 | 0.887 | 0.622-1.264 | 0.506 |
| No |  |  |  |  |
| Senna | 19/56 | Ref. | Ref. | Ref. |
| Lactulose | 8/15 | 1.281 | 0.475-3.456 | 0.624 |
| Polyethylene Glycol | 43/101 | 1.121 | 0.609-2.063 | 0.714 |
| Docusate Sodium | 35/136 | 0.558 | 0.283-1.101 | 0.093 |
| **Initial shockable rhythm** |  |  |  |  |
| Yes |  |  |  |  |
| Senna | 20/88 | Ref. | Ref. | Ref. |
| Lactulose | 12/24 | 1.190 | 0.503-2.816 | 0.692 |
| Polyethylene Glycol | 41/151 | 1.301 | 0.724-2.336 | 0.379 |
| Docusate Sodium | 57/305 | 0.707 | 0.389-1.285 | 0.255 |
| No |  |  |  |  |
| Senna | 40/109 | Ref. | Ref. | Ref. |
| Lactulose | 39/57 | 1.668 | 1.031-2.765 | 0.037 |
| Polyethylene Glycol | 103/207 | 1.456 | 1.000-2.119 | 0.050 |
| Docusate Sodium | 117/502 | 0.925 | 0.640-1.336 | 0.677 |
| **Lay rescuer CPR** |  |  |  |  |
| Yes |  |  |  |  |
| Senna | 2/11 | Ref. | Ref. | Ref. |
| Lactulose | 16/20 | 3.707 | 0.707-19.420 | 0.121 |
| Polyethylene Glycol | 4/10 | 1.434 | 0.203-10.116 | 0.718 |
| Docusate Sodium | 72/171 | 1.749 | 0.367-8.337 | 0.483 |
| No |  |  |  |  |
| Senna | 58/168 | Ref. | Ref. | Ref. |
| Lactulose | 35/61 | 1.369 | 0.860-2.179 | 0.186 |
| Polyethylene Glycol | 140/348 | 1.303 | 0.951-1.784 | 0.099 |
| Docusate Sodium | 162/636 | 0.722 | 0.523-0.996 | 0.047 |

**Continued supplementary table 5.**

| **Subgroup** | **Mortality/total number** | **Hazard ratio** | **95% CI** | ***P*-value** |
| --- | --- | --- | --- | --- |
| **Hypothermia** |  |  |  |  |
| Yes |  |  |  |  |
| Senna | 8/19 | Ref. | Ref. | Ref. |
| Lactulose | 6/12 | 0.763 | 0.193-3.016 | 0.699 |
| Polyethylene Glycol | 23/41 | 1.134 | 0.467-2.753 | 0.782 |
| Docusate Sodium | 40/95 | 0.896 | 0.337-2.381 | 0.825 |
| No |  |  |  |  |
| Senna | 52/178 | Ref. | Ref. | Ref. |
| Lactulose | 45/69 | 1.566 | 0.996-2.462 | 0.052 |
| Polyethylene Glycol | 121/317 | 1.358 | 0.973-1.896 | 0.072 |
| Docusate Sodium | 194/712 | 0.809 | 0.583-1.123 | 0.206 |
| **MCS** |  |  |  |  |
| Yes |  |  |  |  |
| Senna | 6/19 | Ref. | Ref. | Ref. |
| Lactulose | 0/3 | - | - | 0.970 |
| Polyethylene Glycol | 9/19 | 0.942 | 0.193-4.601 | 0.941 |
| Docusate Sodium | 32/80 | 0.318 | 0.067-1.501 | 0.148 |
| No |  |  |  |  |
| Senna | 54/178 | Ref. | Ref. | Ref. |
| Lactulose | 51/78 | 1.705 | 1.120-2.594 | 0.013 |
| Polyethylene Glycol | 135/339 | 1.367 | 0.991-1.886 | 0.057 |
| Docusate Sodium | 202/727 | 0.847 | 0.667-1.164 | 0.306 |
| **Early enteral nutrition** |  |  |  |  |
| Yes |  |  |  |  |
| Senna | 16/37 | Ref. | Ref. | Ref. |
| Lactulose | 22/43 | 1.087 | 0.536-2.202 | 0.817 |
| Polyethylene Glycol | 52/98 | 1.673 | 0.914-3.061 | 0.095 |
| Docusate Sodium | 187/431 | 0.991 | 0.556-1.765 | 0.975 |
| No |  |  |  |  |
| Senna | 44/160 | Ref. | Ref. | Ref. |
| Lactulose | 29/38 | 1.676 | 0.952-2.950 | 0.074 |
| Polyethylene Glycol | 92/260 | 1.149 | 0.793-1.664 | 0.463 |
| Docusate Sodium | 137/554 | 0.693 | 0.477-1.005 | 0.053 |
| **Invasive ventilator** |  |  |  |  |
| Yes |  |  |  |  |
| Senna | 36/125 | Ref. | Ref. | Ref. |
| Lactulose | 24/46 | 1.718 | 0.971-3.037 | 0.063 |
| Polyethylene Glycol | 88/203 | 1.568 | 1.053-2.335 | 0.027 |
| Docusate Sodium | 164/467 | 1.098 | 0.741-1.628 | 0.641 |
| No |  |  |  |  |
| Senna | 24/72 | Ref. | Ref. | Ref. |

**Continued supplementary table 5.**

| **Subgroup** | **Mortality/total number** | **Hazard ratio** | **95% CI** | ***P*-value** |
| --- | --- | --- | --- | --- |
| Lactulose | 27/35 | 1.525 | 0.795-2.927 | 0.205 |
| Polyethylene Glycol | 56/155 | 1.024 | 0.612-1.713 | 0.928 |
| Docusate Sodium | 70/340 | 0.545 | 0.325-0.915 | 0.022 |
| **SOFA** |  |  |  |  |
| ≥7 |  |  |  |  |
| Senna | 34/100 | Ref. | Ref. | Ref. |
| Lactulose | 45/67 | 1.857 | 1.133-3.042 | 0.014 |
| Polyethylene Glycol | 88/179 | 1.591 | 1.054-2.401 | 0.027 |
| Docusate Sodium | 141/357 | 1.048 | 0.695-1.579 | 0.824 |
| <7 |  |  |  |  |
| Senna | 26/97 | Ref. | Ref. | Ref. |
| Lactulose | 6/14 | 1.006 | 0.354-2.864 | 0.991 |
| Polyethylene Glycol | 56/179 | 1.347 | 0.804—2.254 | 0.258 |
| Docusate Sodium | 93/450 | 0.684 | 0.410-1.139 | 0.144 |
| **Vasoactive inotropic score** |  |  |  |  |
| ≥4 |  |  |  |  |
| Senna | 33/72 | Ref. | Ref. | Ref. |
| Lactulose | 40/57 | 1.208 | 0.729-2.002 | 0.463 |
| Polyethylene Glycol | 74/150 | 1.095 | 0.710-1.687 | 0.681 |
| Docusate Sodium | 169/412 | 0.788 | 0.525-1.181 | 0.248 |
| <4 |  |  |  |  |
| Senna | 27/125 | Ref. | Ref. | Ref. |
| Lactulose | 11/24 | 1.872 | 0.830-4.223 | 0.131 |
| Polyethylene Glycol | 70/208 | 1.874 | 1.167-3.011 | 0.009 |
| Docusate Sodium | 65/395 | 0.915 | 0.551-1.517 | 0.729 |

**Supplementary table 6.** Standardized mean differences (SMD) before and after propensity score matching

|  | **Before PSM**  **SMD** | **After PSM**  **SMD** |
| --- | --- | --- |
| Female, n (%) | 0.025 | 0.031 |
| Age, year, median (IQR) | 0.874 | 0.022 |
| BMI, kg/m^2^, | 0.358 | 0.029 |
| Smoke, n (%) | 0.022 | 0.029 |
| Race | 0.046 | 0.048 |
| Comorbidities |  |  |
| Heart failure, n (%) | 0.026 | 0.033 |
| Myocardial infarction, n (%) | 0.025 | 0.032 |
| Stroke^*^, n (%) | 0.117 | 0.022 |
| Diabetes mellitus, n (%) | 0.025 | 0.032 |
| Renal dysfunction, n (%) | 0.023 | 0.030 |
| Charlson Comorbidity Index, median (IQR) | 0.155 | 0.035 |
| OHCA, n (%) | 0.022 | 0.027 |
| Bystander CPR, n (%) | 0.018 | 0.023 |
| Initial rhythm shockable, n (%) | 0.025 | 0.033 |
| ECPR, n (%) | 0.005 | 0.004 |
| Heart rate, bpm, median (IQR) | 0.884 | 0.017 |
| MAP, mmHg, median (IQR) | 0.579 | 0.035 |
| Lactate, mmol/L, median (IQR) | 0.159 | 0.021 |
| pH, median (IQR) | 0.006 | 0.001 |
| Hypothermia, n (%) | 0.061 | 0.023 |
| Invasive ventilator, n (%) | 0.085 | 0.032 |
| Coronary arteriography or percutaneous coronary intervention, n (%) | 0.012 | 0.015 |
| CRRT, n (%) | 0.016 | 0.021 |
| MCS^#^, n (%) | 0.031 | 0.016 |
| Early enteral nutrition^$^, n (%) | 0.059 | 0.029 |
| Medications |  |  |
| Epinephrine, n (%) | 0.077 | 0.022 |
| Norepinephrine, n (%) | 0.026 | 0.033 |
| Dopamine, n (%) | 0.016 | 0.020 |
| Dobutamine, n (%) | 0.019 | 0.025 |
| Milrinone, n (%) | 0.009 | 0.011 |
| Muscle relaxants, n (%) | 0.018 | 0.023 |
| Vasoactive inotropic score, median (IQR) | 0.064 | 0.026 |
| SOFA score, median (IQR) | 0.212 | 0.037 |
| SAPSII score, median (IQR) | 0.822 | 0.035 |
| APSIII score, median (IQR) | 0.653 | 0.032 |
| OASIS score, median (IQR) | 0.460 | 0.019 |

**Supplementary table 7.** Results of Schoenfeld residual analysis for covariates in the Cox regression models

| **Covariate** | **Pearson correlation coefficient** | ***P*-value** |
| --- | --- | --- |
| laxative | 0.205 | <0.001 |
| Female | 0.079 | 0.148 |
| Age, year | 0.073 | 0.186 |
| BMI | -0.051 | 0.356 |
| Smoke | 0.096 | 0.080 |
| Race | 0.026 | 0.641 |
| Comorbidities |  |  |
| Heart failure | 0.049 | 0.374 |
| Myocardial infarction | -0.017 | 0.761 |
| Stroke | -0.028 | 0.612 |
| Diabetes mellitus | -0.027 | 0.628 |
| Renal dysfunction | -0.050 | 0.367 |
| Charlson Comorbidity Index | 0.017 | 0.756 |
| OHCA | -0.090 | 0.102 |
| Bystander CPR | 0.025 | 0.651 |
| Initial rhythm shockable | -0.007 | 0.892 |
| ECPR | 0.096 | 0.079 |
| Heart rate | 0.126 | 0.021 |
| MAP | -0.117 | 0.033 |
| Lactate | 0.053 | 0.336 |
| pH | -0.003 | 0.952 |
| Hypothermia | -0.056 | 0.251 |
| Invasive ventilator | -0.092 | 0.093 |
| CAG/PCI | 0.028 | 0.583 |
| CRRT | 0.070 | 0.200 |
| MCS | 0.056 | 0.311 |
| Early enteral nutrition | 0.063 | 0.287 |
| Medications |  |  |
| Epinephrine | 0.060 | 0.273 |
| Norepinephrine | 0.106 | 0.052 |
| Dopamine | -0.009 | 0.865 |
| Dobutamine | -0.048 | 0.385 |
| Milrinone | 0.025 | 0.644 |
| Muscle relaxants | 0.036 | 0.518 |
| Vasoactive inotropic score | 0.003 | 0.954 |
| SOFA score | 0.066 | 0.227 |
| SAPSII score | 0.106 | 0.053 |
| APSIII score | 0.098 | 0.076 |
| OASIS score | 0.020 | 0.710 |

**Note:** compliance with the proportional hazards (PH) assumption was assessed via bivariate Pearson correlation analysis between the partial residuals of covariates and the rank of the time variable. A resulting *P*<0.05 indicates violation of the PH assumption for that covariate, necessitating further adjustment.
